# Routine Screening for Cushing's Syndrome Is Not Required in Patients Presenting with Obesity

**DOI:** 10.1155/2013/321063

**Published:** 2013-06-11

**Authors:** Serap Baydur Sahin, Hacer Sezgin, Teslime Ayaz, Emine Uslu Gur, Kadir Ilkkilic

**Affiliations:** ^1^Department of Endocrinology and Metabolism Disease, Recep Tayyip Erdogan University Medical School, 53020 Rize, Turkey; ^2^Department of Family Medicine, Recep Tayyip Erdogan University Medical School, Rize, Turkey; ^3^Department of Internal Medicine, Recep Tayyip Erdogan University Medical School, Rize, Turkey

## Abstract

*Background*. Cushing's syndrome (CS) is a relatively unusual condition that resembles many of the phenotypic features of obesity. Our aim was to evaluate the frequency of CS in obese patients. *Materials and Methods*. This study included 354 consecutive patients (87.9% female, age 37.8 ± 13.4 years) who presented with simple obesity. All the patients were evaluated for the clinical signs of CS. Lipid parameters, fasting glucose (FPG) and insulin, 75 gr oral glucose tolerance test, basal cortisol and ACTH were measured. 1 mg overnight DST was performed. *Results*. The mean weight of the patients was 102.4 ± 20.1 kg and BMI 40 ± 7.35 kg/m^2^. 34.5% of the patients were hypertensive. 36.2% of the patients had central obesity, 72% dorsocervical fat accumulation, 28.8% abdominal striae and 23.2% acne. 49.4% of the women had hirsutism. 46.5% had prediabetes and 12.0% had type 2 diabetes, 72.6% had dyslipidemia. The mean cortisol and ACTH levels were as follows: 9.28 ± 3.53 **μ**g/dL and 17.02 ± 10.43 pg/mL. Seven patients failed to suppress plasma cortisol to less than 1.8 **μ**g/dL. Biochemical confirmation tests were performed in these patients and 2 of them were diagnosed glucocorticoid-secreting adrenal adenoma. *Conclusions*. Routine screening for CS in obese patients is not required.

## 1. Introduction

Cushing's syndrome (CS) is considered a contributing factor to the development of obesity. On the other hand, obesity itself might share the symptoms and signs of CS such as hirsutism, menstrual abnormalities, acne, dorsocervical fat pad (buffalo hump), supraclavicular fullness, glucose intolerance, and hypertension. Physicians may be called upon to exclude CS in obese patients, who are increasingly present in the general population. Early recognition of CS can reduce the morbidity and mortality [1]. 

The diagnosis of CS is often a challenge for clinicians due to the variable pattern and the nonspecificity of clinical manifestations. The diagnosis can be difficult particularly in states of mild or cyclical or periodical hypercortisolism [2–4]. The suspicion of CS arises in the presence of concomitant recent weight gain, impaired glucose tolerance, and high blood pressure [3]. Several studies reported a 1–5% prevalence of unsuspected CS in patients with poorly controlled type 2 diabetes and/or hypertension [5–8]. However, there are only few studies on the prevalence of CS in obese patients [9–13]. We therefore aimed to evaluate the frequency of CS in patients who present with obesity.

The 1 mg overnight dexamethasone-suppression test (DST) is the most frequently used screening tool for CS [14]. Because it is easy to perform and has low cost it is used as a first-line screening test in outpatient screening. However, the lack of suppression after 1 mg overnight DST may be seen in 2–8% of the obese individuals [10, 11, 14]. Our second aim was to reevaluate the validity of the 1 mg overnight dexamethasone suppression test as a CS screening test in obese patients.

## 2. Patients and Methods

This study included 354 consecutive patients with a body mass index (BMI) >30 kg/m^2^ who were admitted to our endocrine outpatient because of simple obesity between November 2012 and May 2013. Patients were excluded from the study if they had clear cushingoid features. The other exclusion criteria were exogenous glucocorticoid intake, serious medical conditions that might alter pituitary-adrenal function, factors known to influence the dexamethasone suppression test (drug use such as antiepileptics, estrogens, alcohol dependence, depression, and other psychiatric conditions or pregnancy), and renal failure (creatinine clearance < 30 mL/min). 

All the patients were evaluated for the clinical signs of CS such as central obesity, dorsocervical fat pad, supraclavicular fullness, abdominal striae, facial plethora, moon face, acne, and hirsutism. Weight, height, waist circumference, and systolic and diastolic blood pressure were measured. The BMI was calculated by dividing the body weight in kilograms by the square of the height in meters. 

Blood samples were obtained from all subjects to test the levels of fasting glucose, insulin, lipid parameters (total cholesterol, LDL, HDL cholesterol, and triglyceride), urea, creatinin, free triiodothyronine (fT3) (N: 1.71–3.71 pg/mL), free thyroxin (fT4) (N: 0.93–1.77 ng/dL), and thyrotropin (TSH) (N: 0.15–3.7 IU/mL). HbA1c was measured in known diabetics; all others underwent 75 gr oral glucose tolerance tests. An insulin resistance score, Homeostasis Model Assessment-Insulin resistance (HOMA-IR), was computed by the following formula [15]: HOMA-IR = FPG (mg/dL) × immunoreactive insulin (IRI) (*μ*IU/mL)/405.

Basal levels of adrenocorticotropic hormone (ACTH) (N: 0–46 pg/mL) and cortisol (N: 5–28 *μ*g/dL) were measured in the morning at 0800 h. All patients subsequently underwent a 1 mg overnight DST. The overnight low-dose (1 mg) DST consists of the oral intake of 1 mg dexamethasone between 2300 and 2400 h, followed by measurement of fasting plasma cortisol between 0800 and 0900 h the following morning. Suppression of serum cortisol to <1.8 *μ*g/dL after dexamethasone administration was the cut-off point for normal suppression. Patients whose serum cortisol after 1 mg overnight DST was >1.8 *μ*g/dL underwent a low-dose dexamethasone suppression test (LDDST) as follows: patients started taking 0.5 mg dexamethasone every 6 hours for 2 days; 6 hours after the last dose of dexamethasone, cortisol was measured. Suppression of morning serum cortisol to <1.8 *μ*g/dL was defined as normal suppression. Those who failed to suppress on a LDDST underwent more comprehensive studies to evaluate and localize the source of the hypercortisolism.

### 2.1. Statistical Analysis

SPSS version 19.0 software (SPSS, Chicago, IL, USA) was used for statistical analysis. Results were expressed as mean ± standard deviation. 

## 3. Results

354 consecutive obese patients were screened for CS. Their mean ± SD age was 37.8 ± 13.4 years and 87.9% were female. The mean weight and BMI of the patients were 102.4 ± 20.1 kg and 40 ± 7.35 kg/m^2^ (Table 1). The distribution of BMI was 30–34.9 kg/m^2^, 25.1%; 35–39.9 kg/m^2^, 31.1%; and ≥40 kg/m^2^, 43.8%. 

The most common physical examination findings were dorsocervical fat accumulation (72%) and hirsutism (49.4%). 28.8% of the patients had purple stretch marks in wide of <1 cm (Table 1). 34.5% of the patients were hypertensive. 43 of 354 evaluable subjects (12%) had diabetes and 46.5% had prediabetes. 72.6% of the patients had dyslipidemia. Laboratory findings of the patients are summarized in Table 2. 

Seven patients failed to suppress plasma cortisol less than 1.8 *μ*g/dL after the 1 mg overnight DST. There was no correlation between the cortisol levels after the 1 mg DST and BMI. These patients underwent a confirmatory LDDST and five patients suppressed plasma cortisol less than 1.8 *μ*g/dL and were considered false positives of the 1 mg overnight DST. The post-1 mg DST plasma cortisol in these patients was 2.86 *μ*g/dL (range, 1.9–4.6).

The remaining 2 patients (0.5%), who had a post-1 mg-DST plasma cortisol of 3.15 *μ*g/dL, were further evaluated and plasma cortisol levels were detected greater than 1.8 *μ*g/dL following the LDDST (3.25 *μ*g/dL). Both of them had undetectable ACTH levels (<5 pg/mL) and elevated midnight cortisol. 24-hour urinary free cortisol levels were in normal range. One of the patients was a 39-year-old woman and physical examination revealed central obesity (BMI = 40 kg/m^2^), a buffalo hump, moon facies, and supraclavicular fullness. She had no muscle weakness, no facial plethora, and abdominal striae. She had menstrual irregularities and prediabetes. Computed tomography (CT) scanning of the adrenals identified the cortical adenoma measuring 38 mm on the right side. She underwent a right laparoscopic adrenalectomy. The other patient was a 76-year-old, postmenopausal woman who had central obesity (BMI = 35 kg/m^2^), a 15-year history of type 2 diabetes mellitus, and a 20-year history of hypertension. The patient was being treated with insulin (glycosylated hemoglobin [HbA1c] was 7%), and her hypertension was being controlled with an angiotensin-converting enzyme inhibitor. Computed tomography (CT) scan of the abdomen detected a low-density adrenal mass measuring 3 cm on the right side. She declined to undergo surgical intervention. 

## 4. Discussion

Among 354 people who were obese, two patients (0.5%) were diagnosed with Cushing's syndrome. The false positive rate for the 1 mg overnight dexamethasone-suppression test was 1.4%, even when using a cut-off serum cortisol of 1.8 *μ*g/dL. 

The suspicion of CS depends largely on individual clinical judgment and personal practice. The patients do not always present a clear Cushing phenotype. They can have only the mild signs of hypercortisolism, such as facial fullness and central obesity. It may be difficult to decide whether these signs may be attributable to an underlying occult hypercortisolism or are manifestations of the obesity. In our study, the symptoms or signs for CS, such as facial fullness, dorsocervical fat accumulation, and hirsutism were present in a major proportion of the population. 

Screening for Cushing's syndrome is recommended in patients with multiple and progressive clinical features (facial plethora, easy bruising, striae, and proximal myopathy), patients with unusual features for age (e.g., osteoporosis, hypertension, and type 2 diabetes), and patients with adrenal incidentaloma by the Endocrine Society guidelines [16]. Early recognition of patients with CS, particularly those with milder forms, is important to prevent the long-term physical consequences and increased mortality that may occur when the disease is left untreated [17]. Therefore we screened the obese patients for CS who have not a clear Cushing phenotype. In our study population, which includes morbidly obese patients in major proportion, mean age of the patients was young and also the prevalence of glucose intolerance and hypertension was high. 

The reported prevalence of CS among the obese patients varies widely between the different studies, ranging from 0.6% to 9.4% [10, 11, 13, 14]. This heterogeneity may be due to the different inclusion criteria as well as the different cut-off values to define cortisol suppression after the 1 mg DST. Janković et al. screened 433 morbidly obese patients and found the prevalence of CS below 0.6% [13]. They used the 1 mg-DST for screening CS, but defined the cut-off value as 3 *μ*g/dL differently from our study. In the other three studies, CS was screened in a small number of patients [10, 11, 14]. Tiryakioglu et al. demonstrated a high prevalence of CS in the obese population despite the absence of other signs or symptoms of the disorder (9.4%). The cutoff value for the 1 mg DST was 1.8 *μ*g/dL [10]. 

Some studies evaluated the prevalence of occult CS in overweight and obese patients with uncontrolled diabetes and it was found to be 0–9.4% in different studies [5, 15, 18, 19]. In all the studies, the first screening step was performed with the 1 mg DST, but the cutoff values for the suppression of cortisol were different (from 1.8 to 5 *μ*g/dL).

The 1 mg overnight dexamethasone suppression test is proved to be a simple, sensitive, and highly specific screening test for Cushing's syndrome. Serum cortisol after overnight 1 mg DST above 50 nmol/L (18 ng/mL or 1.8 mg/dL) is considered to be suggestive of Cushing's syndrome [16]. 24 h urine cortisol, 1 mg overnight DST and midnight cortisol, and combined strategies based on these tests have similar accuracy [16, 20]. 2 mg 48 h dexamethasone suppression test is considered a second-line test because it is not often simple to carry out in an outpatient [16].

The lack of suppression after 1 mg overnight DST may be seen in the obese individuals. The ratio of false positive 1 mg DST results in obese population was as follows in the different studies: 8% [11] and 2.3% [12]. In our study, this ratio was 1.4%. Our results suggest that even in the obese population, 1 mg overnight DST is a sensitive and specific screening test for Cushing's syndrome. 

In conclusion, the present data do not support widespread screening of obese patients for Cushing's syndrome. We suggest that examination for hypercortisolism should only be performed in obese patients with a cushingoid appearance and hypertension or glucose intolerance or dyslipidaemia.

## Figures and Tables

**Table 1:** Demographic and clinical features of the patients.

	Patients (*n* = 354)
Age (years)	37.8 ± 13.4
Gender female (%)	87.9%
BMI (kg/m^2^)	40 ± 7.35
Waist circumference (cm)	114.62 ± 14.15
Systolic blood pressure (mmHg)	136.76 ± 18.6
Diastolic blood pressure (mmHg)	80.5 ± 11.9
Presence of central obesity (%)	36.2%
Dorsocervical fat accumulation (%)	72%
Acne (%)	23.2%
Hirsutism (females) (%)	49.4%
Acanthosis nigricans (%)	31.4%
Abdominal striae (%)	28.8%
Supraclavicular fullness (%)	19%
Facial plethora (%)	15%

Values are expressed as means ± SD. BMI: body mass index.

**Table 2:** Laboratory findings of the patients.

	Patients (*n* = 354)
Basal cortisol (*µ*g/dL)	9.28 ± 3.53
ACTH (pg/mL)	17.02 ± 10.43
Free T3 (pg/mL)	3.03 ± 0.6
Free T4 (ng/dL)	1.3 ± 4.2
TSH (uIU/mL)	2.44 ± 3.7
Fasting plasma glucose (mg/dL)	112.49 ± 46.59
HOMA-IR	3.11 ± 2.03
Total cholesterol (mg/dL)	215.5 ± 42.1
Triglyceride (mg/dL)	154.41 ± 82.8
LDL cholesterol (mg/dL)	139.86 ± 6.3
HDL cholesterol (mg/dL)	45.37 ± 11.1

Values are expressed as means ± SD.
